# Stent Graft–Induced New Entry Created at Locations Other Than Both Proximal and Distal Ends

**DOI:** 10.1016/j.jaccas.2025.105162

**Published:** 2025-09-24

**Authors:** Satoki Nakamura, Tomoaki Kudo, Junki Yokota, Masatoshi Hata, Toru Kuratani

**Affiliations:** Department of Cardiovascular Surgery, Osaka International Medical and Science Center, Osaka, Japan

**Keywords:** aortic dissection, endovascular aortic repair, stent graft-induced new entry

## Abstract

One of the complications after thoracic endovascular aortic repair (TEVAR) is stent graft–induced new entry (SINE). SINE occurs in 5% to 25% of aortic dissection cases treated with TEVAR and is thought to be associated with endograft oversizing and springback force. Although SINE typically occurs at the proximal or distal ends of the endograft, we report a rare case of managing a SINE formation within the main body of the stent graft, distinct from its landing zones. This unusual presentation highlights the importance of recognizing atypical SINE mechanisms and locations, which can lead to significant complications such as endoleak.

**Take-Home Messages:**

To prevent SINE after TEVAR, it is crucial to consider not only the landing zone and device sizing but also the entire aortic curvature where the stent graft will be deployed. Careful device selection and procedural planning are required.

## Graphical Abstract

**Figure:**
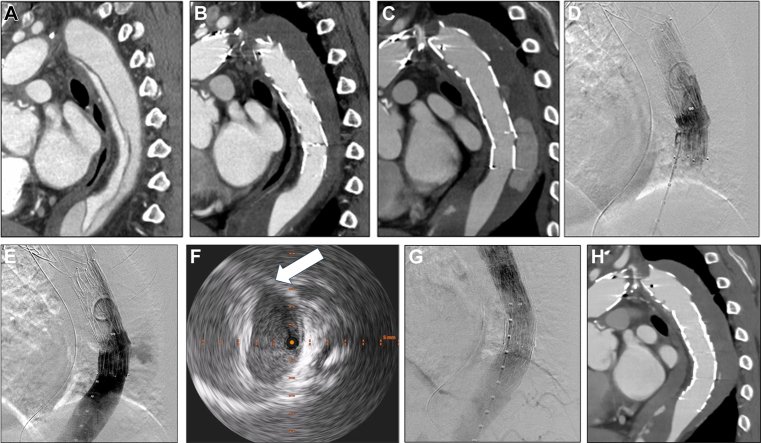

A 75-year-old man presented to our hospital with acute-onset chest and back pain. He had a total arch replacement for acute type A aortic dissection and a thoracic endovascular aortic repair (TEVAR) (Valiant, Medtronic; and TXD, Cook Medical) for a residual type B aortic dissection at another institution 6 and 5 years ago, respectively (Figures 1A and 1B). According to computed tomography angiography (CTA) findings, we suspected: 1) distal stent graft–induced new entry (SINE); 2) SINE and type 3b endoleak (stent graft fabric rupture); or 3) SINE and type 1b endoleak (Figure 1C). It was not easy to differentiate between these 3 possibilities. However, the treatment plan involved stent graft relining and extension to the distal side.

**Take-Home Messages**

• To prevent SINE, it is crucial to consider not only the landing zone and device sizing but also the entire aortic curvature where the stent graft will be deployed.
• Careful device selection and procedural planning are required.

**Figure 1:**
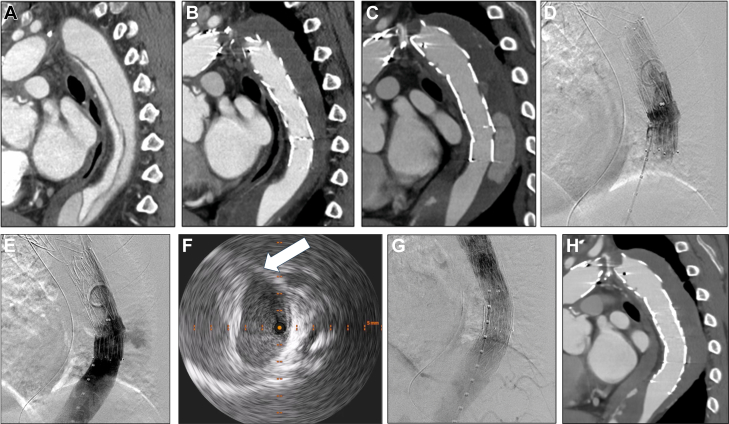
Preprocedure and Postprocedure Computed Tomography Angiography, Intraoperative Angiography, and Intravascular Ultrasound (A) No re-entry at the descending aorta after total arch replacement. (B) No endoleaks 5 years ago. (C) Endoleak at the time of visiting the hospital. (D) No type 3 endoleak. (E) No distal stent graft–induced new entry. (F) Defect of aortic wall (arrow). (G) No endoleak. (H) No endoleak.

Intraoperative angiography showed no evidence of type 3b endoleak or distal SINE (Figures 1D and 1E). Additionally, the intravascular ultrasound revealed a 20-mm defect of the aortic wall located 30 mm distal to the stent graft, as well as a low-density area in the false lumen (Figure 1F, Video 1). Based on these findings, we diagnosed that the exoskeleton of the stent graft inserted 5 years ago had protruded into the false lumen on the greater curvature side from all but both ends, forming a SINE, which had changed the placement site of the distal stent graft and resulted in a type 1b endoleak (Figure 1D, Video 2). To address this issue, we performed an additional TEVAR (CTAG, W.L. Gore & Associates). Postprocedure angiography and postoperative CTA confirmed the resolution of both type 1b endoleak and inflow of contrast medium into the false lumen (Figures 1G and 1H). The patient was discharged home on postoperative day 7.

It is widely recognized that close attention to several factors, including precise sizing of both proximal and distal landing zones, appropriate device selection (considering the type of device and the degree of oversizing), and careful determination of the deployment site, with caution against placing the graft in highly curved aortic segments, is crucial.^1^ During this patient's TEVAR procedure 5 years ago, the endograft was inadequately placed, resulting in continued mechanical stress on the aortic wall. This persistent mechanical irritation is considered to have gradually damaged the fragile dissected intima over time, ultimately leading to the formation of SINE. This case highlights the importance of not only accurate stent graft sizing and appropriate device selection but also meticulous deployment technique.

## Discussion

What was the primary cause for the occurrence of SINE at an unusual location in this presented case?

(1) Insufficient blood pressure control for the patient after TEVAR.
(2) Inappropriate stent sizing relative to the true lumen.
(3) Inadvertent stent deployment without conforming to the aortic curvature.
(4) Inaccurate measurement of the landing zone.

The correct answer is (3). The uniqueness of this case lies in the SINE occurring within the main body of the stent graft, precisely where the distal stent was inadvertently positioned in a curved segment, rather than at the more commonly observed proximal or distal landing zones.

Insufficient blood pressure control (1) can predispose to dissection but is not the primary direct cause of SINE. Although inappropriate stent sizing (2) and inaccurate measurement of the landing zone (4) are known risk factors of SINE and endoleak, the distinct feature of CTA in this case is the SINE originating from the graft body. In particular, oversizing (2) is generally associated with the development of distal SINE.

## Funding Support and Author Disclosures

The authors have reported that they have no relationships relevant to the contents of this paper to disclose.

## Appendix

Video 1 Intravascular Ultrasound Reveals a 20-mm Linear Defect in the Aortic Wall Located Approximately 30 mm Distal to the Stent Graft A low-density area is observed in the adjacent false lumen, suggesting communication between the true and false lumens via the defect, indicating a stent graft–induced new entry (SINE).

Video 2 Intraoperative Angiography Shows Contrast Medium Entering the False Lumen From the Midportion of the Previously Implanted Stent Graft Rather Than From Either End This finding supports the diagnosis of a nonterminal SINE that caused stent graft misalignment and a subsequent type 1b endoleak.
